# Clinical outcomes of combined presbyopia and astigmatism correction following hydrophilic trifocal toric intraocular lens implantation in cataract patients: a prospective clinical study

**DOI:** 10.1186/s12886-026-05145-5

**Published:** 2026-07-28

**Authors:** Péter Dormán, Zoltán Zsolt Nagy

**Affiliations:** Department of Ophthalmology, Faculty of Medicine, Semmelweis University, Mária utca 39, Budapest, 1085 Hungary

**Keywords:** Cataract surgery, Presbyopia, Intraocular lens, Trifocal toric IOL, Corneal astigmatism, Rotational stability, Liberty 677MTY, Full Range of Field, FRoF-Sm, Functional classification

## Abstract

**Background:**

Presbyopia and corneal astigmatism frequently coexist in cataract patients, reducing visual quality and increasing spectacle dependence. Toric trifocal intraocular lenses (IOLs) aim to address both conditions simultaneously. This prospective study evaluated the visual performance, quality, patient satisfaction, and safety profile of the Liberty 677MTY toric IOL, and classified its functional profile based on distance-corrected monocular defocus curve analysis.

**Methods:**

This prospective, single-center, non-comparative clinical investigation included 28 eligible cataract patients with corneal astigmatism (1.00–6.00 D) who underwent bilateral implantation of the Liberty 677MTY trifocal toric IOL. Uncorrected and distance-corrected visual acuities at all distances, subjective refraction, IOL rotational stability, defocus curves, contrast sensitivity, patient-reported visual function, and safety were reported during a 12-month follow-up period.

**Results:**

The Liberty 677MTY IOL demonstrated significant improvements (*p* < 0.05) in uncorrected and distance-corrected visual acuities and subjective refractive cylinder, with stability over time. IOL alignment was stable through follow-up, with mean signed and absolute rotations of < 2° and < 5°, respectively. Binocular visual acuity defocus curves showed visual acuities better than 0.2 logMAR across the range of + 1.00 to ‑3.50 D. Monocular visual acuity defocus analysis confirmed full range of field smooth (FRoF-Sm) classification of the IOL. Contrast sensitivity defocus curve met acceptance criteria. Based on patient-reported outcomes 89% rated satisfaction as high, and 83% reported mild or no difficulty with visual disturbances. At 12 months, spectacle independence was achieved in 96.3% of patients for far, 81.5% for intermediate, and 92.6% for near vision. Safety analysis revealed one Nd: YAG-treated PCO case (1.61%) and no other IOL-related adverse events.

**Conclusions:**

This study provides a detailed evaluation of the Liberty 677MTY trifocal toric IOL, integrating real-life binocular visual experience with comprehensive monocular assessments. The lens demonstrated predictable refractive outcomes, excellent rotational stability, and consistently high-quality vision across all distances, with strong patient satisfaction. Classified as FRoF-Sm by monocular defocus curve analysis, the lens offers continuous functional vision. By integrating monocular and binocular perspectives, these findings offer a clearer understanding of the lens’s performance, supporting its use as a safe and effective solution for achieving spectacle independence and enhancing postoperative quality of life.

## Background

Presbyopia, the age-related decline in accommodative ability, affects nearly all individuals over the age of 45, significantly impairing near vision, thereby reducing quality of life and increasing dependence on spectacles or other visual aids [1]. Astigmatism, caused by curvature differences in the corneal surface, results in unequal refractive power across the two principal meridians of a lens, and results in blurred vision [2]. Preoperative corneal astigmatism of ≥ 1.00 diopter (D) is present in approximately 35–45% of cataract patients [3, 4], and residual astigmatism > 0.50 D after multifocal intraocular lens (IOL) implantation has been shown to negatively impact visual outcomes, particularly at far and intermediate distances [5].

Toric trifocal IOLs are designed to simultaneously treat presbyopia and postoperative corneal astigmatism, providing patients with the potential for spectacle independence across all visual ranges. Published clinical studies of presbyopia-correcting IOLs frequently prioritize binocular functional endpoints and patient-reported outcomes because these reflect real-world performance (e.g., spectacle independence and dysphotopsias). However, current standards and measurement frameworks emphasize monocular characterization, which can provide insight into the true optical performance of the lens itself. Accordingly, the present study evaluates the clinical performance, visual function, and patient satisfaction associated with the implantation of Liberty 677MTY trifocal toric IOL (Medicontur Medical Engineering Ltd, Hungary) in combination with detailed monocular performance data, including visual acuity (VA), contrast sensitivity, and rotational stability, to provide a more complete characterization of the lens. In 2024, a new functional classification system for multifocal IOLs was introduced to better differentiate lens performance characteristics [6]. Our study aims to position the Liberty 677MTY within this framework, based on the assessment of the distance-corrected monocular defocus curve.

Additionally, since the Liberty 677MTY is a hydrophilic acrylic lens, its long-term safety profile remains a topic of clinical interest. This study also aimed to investigate the safety profile of Liberty lens when implanted in patients without elevated risk for posterior capsule opacification (PCO). Consequently, pre-existing conditions known to increase the risk of PCO, such as uveitis [7], and diabetic retinopathy [8] were defined as exclusion criteria.

## Materials and methods

### Study design

This was a prospective, observational, non-comparative, single-center clinical investigation conducted at the Department of Ophthalmology, Semmelweis University, Budapest, Hungary. The study adhered to the tenets of the Declaration of Helsinki and received approval from the National Scientific and Ethical Committee of the Medical Research Council of Hungary and the National Institute of Pharmacy and Nutrition, Hungary (OGYÉI/69051/2019).

### Patient selection

Thirty-one cataract patients with preoperative corneal astigmatism requiring cylindrical correction between 1.00 and 6.00 D were screened and provided written informed consent. Twenty-eight patients met the inclusion criteria and were included in the performance analysis. Exclusion criteria included irregular astigmatism, congenital eye abnormalities, prior ocular surgery or trauma, and coexisting ocular pathologies (amblyopia, glaucoma, severe retinal diseases, corneal diseases, uveitis, high myopia). Significant systemic conditions were also grounds for exclusion.

### Surgical technique and investigational device

All surgeries were performed by the same surgeon (Z.Zs.N.) using standard phacoemulsification technique with clear corneal incision under topical anesthesia. Bilateral cataract surgeries were performed on separate days. Each eye received a single-piece, hydrophilic acrylic trifocal toric presbyopia- and astigmatism-correcting IOL (Liberty 677MTY; Medicontur Medical Engineering Ltd, Hungary) with + 1.75 D intermediate and + 3.50 D near addition. A 5 mm capsulorhexis was created during all surgeries, and Amvisc ophthalmic viscosurgical device (OVD) (Bausch & Lomb, Canada) was used. Preoperative biometry was performed using Lenstar LS 900^®^ (Haag-Streit AG, Switzerland) or the IOLMaster 700 (Carl Zeiss Meditech, Germany). IOL power calculations were based on the Barrett Universal II and the Medicontur IOL Optimizer (Medicontur Medical Engineering Ltd, Hungary) formulas, incorporating a 110° incision position and 0.33 D surgically induced astigmatism. Toric alignment was guided intraoperatively by the Verion system (Alcon, USA).

### Outcome measurements

Patients were assessed preoperatively, on the day of surgery, and at scheduled postoperative visits up to 12 months.

Preoperative assessments included optical biometry (keratometry: K1, K2; axial length: AXL; anterior chamber depth: ACD), slit-lamp biomicroscopy, fundoscopy, macular optical coherence tomography (OCT), tonography, tear film stability, subjective refraction (spherical: SPH; and cylindrical: CYL), and monocular distance visual acuities (UDVA, CDVA).

Postoperatively, monocular and binocular visual acuities were measured as uncorrected and corrected distance (UDVA, CDVA, at 4 m), uncorrected and distance-corrected intermediate (UIVA, DCIVA, at 66 cm), and uncorrected and distance-corrected near visual acuity (UNVA, DCNVA at 40 cm). Subjective refraction, including spherical and cylindrical components, was recorded at each visit.

IOL alignment was documented intraoperatively using the Alcon Verion system, and postoperatively by slit-lamp photography at 1, 3, and 12 months. Rotational stability was reported as axis changes, which were expressed as clockwise (negative) or counterclockwise (positive) rotation relative to the intraoperative alignment.

Monocular and binocular distance-corrected visual acuity defocus curves (VADC), and binocular contrast sensitivity defocus curves (CSDC) were plotted at 12 months, using the Multifocal Lens Analyzer (MLA) (Qvision, Spain) application on iPad devices (Apple Inc., USA). Both measurements were performed under photopic conditions, with screen luminance set to 73% and ambient room illumination maintained at 300 lx. To quantify lens performance, area under the curve (AUC) values were calculated for total (AucT), far (AucF), intermediate (AucI) and near (AucN) defocus distances above the 0.3 logMAR threshold for visual acuity, and 0.3 logCS (representing 50% contrast) for contrast sensitivity. According to the MLA’s predefined defocus segmentation, the following ranges were used for AUC calculations: total (TAUC) from + 1.00 D to − 4.00 D in 0.50 D steps, far (FAUC) from + 0.50 D to − 0.50 D, intermediate (IAUC) from − 0.50 D to − 2.00 D, and near (NAUC) from − 2.00 D to − 4.00 D.

Contrast sensitivity was measured monocularly and binocularly using the CSV-1000 (VectorVision, USA) under both photopic and mesopic conditions, and the results were evaluated by comparison to the normal ranges advised by VectorVision (https://www.vectorvision.com/csv1000-norms/).

Patient-reported visual function was assessed using a customized Visual Functioning Questionnaire (VFQ) designed to capture visual symptoms and functional difficulties relevant to multifocal and toric IOL implantation. In addition to general aspects of visual performance, the questionnaire includes targeted items addressing glare and flare, halos, night vision, distorted and double vision, and their impact on daily activities such as night driving and near and distance tasks. These domains are consistent with those assessed in established visual function and symptom-focused instruments, including the NEI-VFQ-25, the Vision and Night Driving Questionnaire (VND-Q), and cataract- and IOL-specific patient-reported outcome measures that emphasize dysphotopsias and real-world visual function. Responses were recorded using a 1–6 point difficulty scale, in line with visual task difficulty assessment frameworks, to allow descriptive evaluation of patient-perceived visual performance alongside objective clinical outcomes. Safety was assessed by monitoring and documenting all IOL-implantation related adverse events during the study.

### Statistical analysis

Data was collected at baseline and all postoperative visits up to 12 months. All analyses were performed using Microsoft Excel (Microsoft Inc., USA) and GraphPad Prism statistical analysis software (GraphPad Software Inc., USA). Visual acuity defocus curves, contrast sensitivity defocus curves, and contrast sensitivity values measured by CSV-1000 were visualized using the same software. Descriptive statistics (mean, standard deviation (SD), median, minimum, maximum) were calculated for continuous variables. Normality was assessed using the D’Agostino & Pearson or Shapiro-Wilk tests.

Paired comparisons were performed using either the two-tailed paired t-test or, when normality assumptions were not met, the Wilcoxon matched-pairs signed-rank test.

For multiple group comparisons, two-way analysis of variance (ANOVA) tests with appropriate post-hoc tests was applied. During the statistical plan making α was set to 0.05, and β at 20%, resulting in an overall statistical power of 80%. Categorical variables were summarized using frequency counts and percentages.

## Results

### Patient demographics

A total of 31 patients were screened and consented to the clinical investigation. Inclusion criteria were met by 28 subjects, who were subsequently evaluated in the performance analysis (Table 1). One patient discontinued participation before the 12-month visit due to a serious adverse event (optic nerve head oedema), which was deemed unrelated to the study procedure or device. The cohort included 21 female (75.0%) and 7 male (25.0%) patients.

**Table 1:** Summary of patient demographics and preoperative characteristics

Baseline characteristics	Mean ± SD	[min, max]
Age (years)	56.59 ± 7.51	[45, 75]
Axial length (mm)	23.43 ± 1.42	[21.16, 28.08]
Anterior chamber depth (mm)	3.18 ± 0.36	[2.09, 3.94]
Average corneal power (D)	43.78 ± 1.42	[40.57, 46.44]
Anterior corneal astigmatism (D)	1.54 ± 1.26	[0.29, 4.73]
Photopic pupil (mm)	2.72 ± 0.30	[2.20, 3.50]

### Functional performance

*Visual Acuity* Monocular visual acuities were measured preoperatively and at 3 and 12 months after the surgery (Table 2). Significant improvements (*p* < 0.0001) in both monocular UDVA and CDVA were observed during the two post-operative visits compared with baseline.

**Table 2:** Uncorrected and distance-corrected monocular visual acuities (Mean ± SD, *p < 0.0001)

Monocular visual acuity (logMAR)	Baseline	3 months follow-up	12 months follow-up
UDVA	0.63 ± 0.44	0.05 ± 0.06 *	0.06 ± 0.06*
CDVA	0.11 ± 0.20	-0.01 ± 0.04 *	0.01 ± 0.03*
UIVA	N/A	0.16 ± 0.12	0.14 ± 0.09
DCIVA	N/A	0.08 ± 0.11	0.08 ± 0.05
UNVA	N/A	0.11 ± 0.09	0.12 ± 0.08
DCNVA	N/A	0.07 ± 0.07	0.07 ± 0.07

**Fig. 1:**
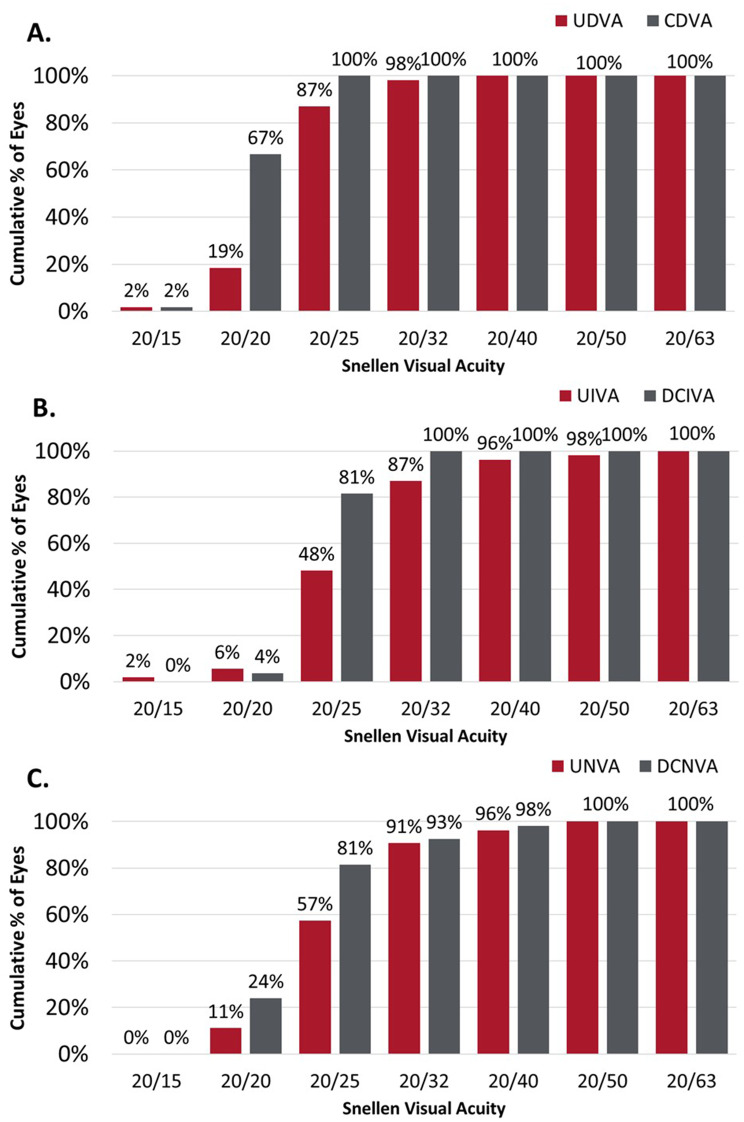
Cumulative Monocular Visual Acuity, for (**A**) Distance, (**B**) Intermediate and (**C**) Near distances. The bar charts illustrate the cumulative percentage of eyes (N=54) achieving specified Snellen visual acuity levels at 12 months postoperatively

At month 12, 100% of patients had a monocular CDVA of ≤ 0.2 logMAR (≤ 20/32 Snellen). 100% of patients also reached this clinically significant VA for DCIVA, and 93% of patients for DCNVA (Fig. 1).

Binocular visual acuities were taken at 3 and 12 months (Table 3). In postoperative examinations, visual acuity results were stable in almost all cases, with statistically significant differences observed in binocular UIVA results (*p* = 0.048).

**Table 3:** Uncorrected and distance-corrected binocular visual acuities (Mean ± SD, *p < 0.05)

Binocular visual acuity (logMAR)	3 months follow-up	12 months follow-up
UDVA	0.00 ± 0.06	0.00 ± 0.05
CDVA	-0.04 ± 0.05	-0.04 ± 0.05
UIVA	0.12 ± 0.08	0.09 ± 0.07*
DCIVA	0.06 ± 0.08	0.04 ± 0.05
UNVA	0.07 ± 0.09	0.07 ± 0.08
DCNVA	0.03 ± 0.07	0.04 ± 0.07

*Postoperative refraction* The mean preoperative spherical equivalent refraction (SE) of the patients was -0.28 ± 3.70 D. At 3 months, 96% of eyes were within 0.50 D and 98% within 1.00 D of emmetropia. At 12 months, 91% were within 0.50 D and 100% within 1.00 D.

Preoperative mean subjective refractive cylinder (CYL) was -1.19 ± 0.88 D. At 3 months, 78% of eyes were within ± 0.50 D and 94% within ± 1.00 D cylinder. At 12 months, 81% of eyes were within ± 0.50 D and 100% within ± 1.00 D cylinder. Manifest cylinder values showed statistically significant improvement by comparing 12-month results to baseline values (*p* < 0.0001).

**Fig. 2:**
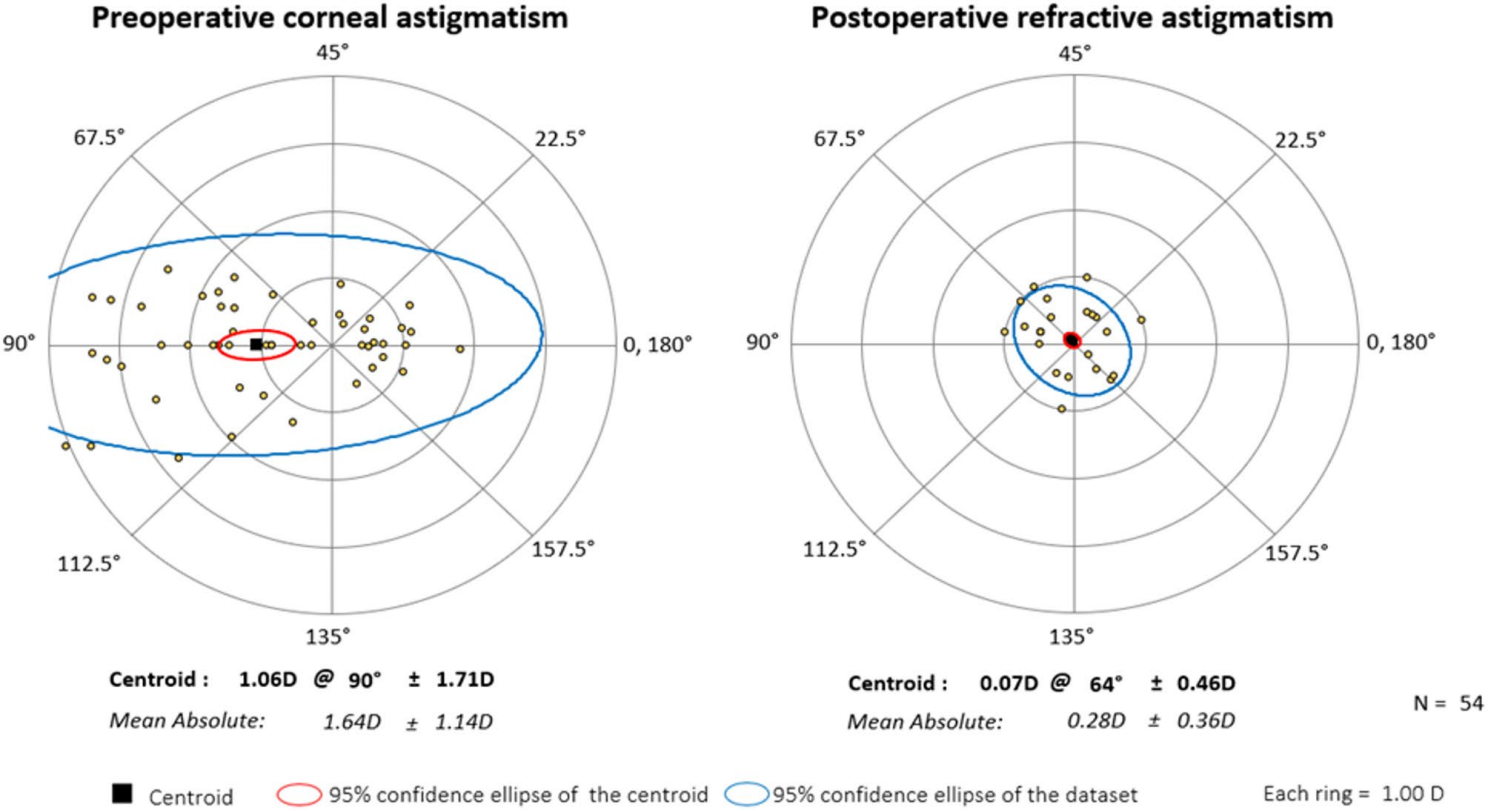
Preoperative and postoperative astigmatism at the corneal plane. Double-angle vector plots illustrate the distribution of preoperative (left plot) and postoperative (right plot) astigmatism (N=54 eyes). In both plots, the black square represents the centroid, the red ellipse denotes the 95% confidence ellipse of the centroid, and the blue ellipse indicates the 95% confidence ellipse of the dataset. Each concentric ring represents 1.00 D of astigmatism

The double-angle plots showing the efficacy of corneal astigmatism correction with 677MTY are presented in Fig. 2.

*Rotational stability* Mean signed rotation was below 2° and mean absolute rotation was below 5° during the 12-month follow-up (Table 4). No repositioning surgeries were needed during the study.

**Table 4:** Signed and absolute rotations over time

Rotation [°]	Follow-up	Mean ± SD
Signed	Month 1	1.50° ± 4.78
	Month 3	1.78° ± 5.48
	Month 12	1.63° ± 5.72
Absolute	Month 1	3.88° ± 3.13
	Month 3	4.70° ± 3.27
	Month 12	4.93° ± 3.27

**Fig. 3:**
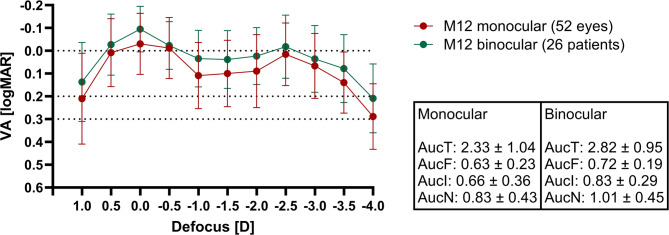
677MTY visual acuity defocus curves at 12 months postoperatively. The graph illustrates the monocular and binocular visual acuity across a range of defocus levels from +1.00 D to -4.00 D. The accompanying table presents the calculated Area Under the Curve (AUC) values, representing the total functional range of vision for both monocular and binocular viewing conditions, at total (AucT), Far (AucF), intermediate (AucI) and near (AucN) distances. N=26 patients, 52 eyes, testing was performed with the Multifocal Lens Analyzer at photopic conditions

*Visual acuity defocus curves* Monocular and binocular distance-corrected visual acuity defocus curves were plotted for 26 patients at 12 months under photopic conditions (Fig. 3).

Both visual acuity defocus curves demonstrated visual acuities of 0.2 logMAR or better across the defocus range from + 1.00 to -3.50 D. Based on monocular defocus curves, the range of field exceeds 3.50 D at 0.2 logMAR, extended to nearly 4.00 D at 0.3 logMAR, and the increase of visual acuity from intermediate to near is 0.08 logMAR.

### Visual quality

**Fig. 4:**
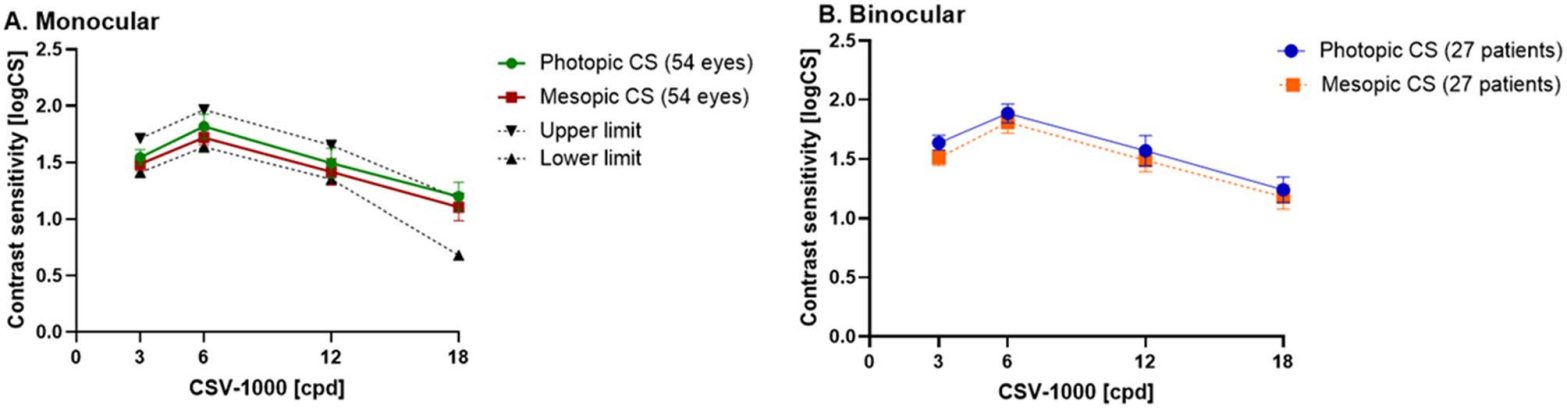
Contrast sensitivity of the 677MTY at 12 months postoperatively. The graph illustrates contrast sensitivity across standard spatial frequencies under both photopic and mesopic conditions. (**A**) Monocular results with upper and lower limits (50–75 years of age) *N* = 54 eyes. (**B**) Binocular contrast sensitivity results *N* = 27 patients. Testing was performed with the CSV-1000 instrument

*Contrast sensitivity* Both monocular and binocular results at 12 months were within the normal reference values defined by VectorVision at all measured frequencies for the 50–75 age group (Fig. 4).

**Fig. 5:**
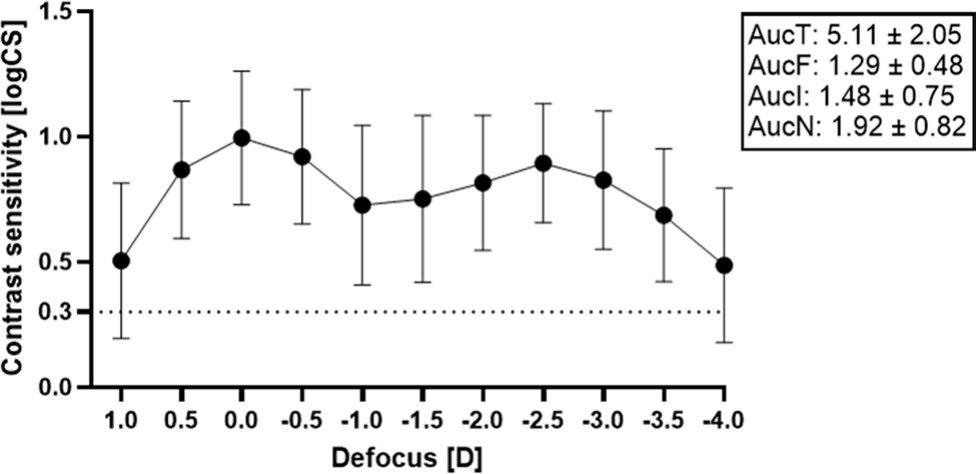
677MTY contrast sensitivity defocus curve at 12 months postoperatively. The graph illustrates the binocular contrast sensitivity across a range of defocus levels from + 1.00 D to -4.00 D. The accompanying table presents the calculated Area Under the Curve (AUC) values, representing the total functional range of contrast at total(AucT), Far (AucF), intermediate (AucI) and near (AucN) distances. *N* = 26 patients, testing was performed with the Multifocal Lens Analyzer at photopic conditions

*Contrast sensitivity defocus curve* To examine contrast sensitivity through the entire defocus range, contrast sensitivity defocus curves were also plotted. The 677MTY trifocal IOL showed peak binocular contrast sensitivity (1.89 ± 0.08 logCS photopic, 1.81 ± 0.09 logCS mesopic) at distance and maintained functional CS from intermediate through near defocus, as reflected by AUC values of 1.29 (far), 1.48 (intermediate), and 1.92 (near), and a total AUC of 5.11 across the entire defocus range (Fig. 5).

*Patient satisfaction* At 12 months, 89% of the patients reported mild or no difficulty with everyday tasks, and 83% reported minimal visual disturbances. Spectacle independence was achieved in 96.3% of patients for far vision, 81.5% for intermediate, and 92.6% for near. Overall, 89% of patients rated their satisfaction with their vision at 8/10 or higher.

*Safety* Safety analyses included all 31 implanted patients (62 eyes). One serious adverse event (optic nerve head oedema, 1.61%) was reported and deemed unrelated to the IOL or procedure. Eight eyes experienced non-serious adverse events with mild or moderate severity (elevated intraocular pressure (IOP) 1.61%, cystoid macular oedema 4.84%, posterior capsule opacification (PCO) 1.61%, Cogan syndrome 3.23%, TASS 1.61%), which all resolved without sequelae. Of these, only one case of PCO was related to the IOL, and this was resolved following Nd: YAG laser capsulotomy.

## Discussion

This study provides one of the first detailed reports on the Liberty 677MTY trifocal toric IOL, combining real-life binocular visual experience with comprehensive monocular assessments. By including monocular outcomes such as visual acuity, contrast sensitivity, and rotational stability, the analysis enables the evaluation of the IOL’s optical performance alongside patient-reported binocular results.

The results of this prospective clinical investigation demonstrate that the Liberty 677MTY IOL provides excellent trifocal optical performance and a favorable safety profile in cataract patients with preoperative corneal astigmatism.

The significant improvement in monocular UDVA and CDVA at 3 and 12 months compared to baseline confirms the effectiveness of the Liberty 677MTY trifocal toric IOL in restoring distance vision. The near-zero logMAR values for UDVA and CDVA at both postoperative visits indicate excellent refractive predictability and stability over time. Intermediate and near visual acuities demonstrated consistently good performance, with minimal differences between uncorrected and distance-corrected conditions, supporting the trifocal design’s ability to provide functional vision across all distances.

Binocular outcomes results represent the visual performance experienced by the patient, with UDVA and CDVA reaching or surpassing emmetropic levels and remaining stable throughout follow-up.

Based on the comparison of the 3-month and 12-month results, the refractive outcomes were found to be highly stable and predictable, with 100% of eyes within ± 1.00 D of target refraction and 91% within ± 0.50 D at 12 months. Subjective refractive cylinder showed significant reduction from baseline, and remained stable throughout the follow-up period. The change of astigmatism between the pre- and post-operative examinations was presented based on the method published by Abulafia et al. [9]. Our results confirm the efficacy of the Liberty 677MTY in correcting refractive errors, confirming previous findings with the same lens model and its non-toric version [10–12].

Rotational stability is a critical factor in the performance of toric IOLs. In this study, the Liberty 677MTY demonstrated excellent rotational stability, with a mean absolute rotation of less than 5° at all postoperative time points, and no cases required surgical repositioning. These findings are consistent with a previous study reporting clinical outcomes after 677MTY implantation [11], reinforcing the rotational stability of the Liberty lens.

The monocular visual acuity defocus curves confirmed that the Liberty 677MTY IOL provides a full range of functional vision, with visual acuity better than 0.2 logMAR across the + 1.00 to − 3.50 D defocus range. Based on monocular defocus curves, the lens achieved a Range of Field (RoF) exceeding 2.30 D at the 0.2 logMAR threshold and greater than 2.75 D at the 0.3 logMAR threshold. The difference in visual acuity between intermediate (0.09 logMAR) and near (0.01 logMAR) was 0.08 logMAR, indicating a smooth transition across focal distances. According to the ESCRS-endorsed functional IOL classification system proposed by Fernández et al. [13], the lens fulfills the criteria for a Full Range of Field - Smooth (FRoF-Sm) profile. Lenses within this subgroup are characterized by a gradual improvement from intermediate to near vision without abrupt drops in visual performance, which is consistent with the observed clinical performance of the Liberty 677MTY. These findings are further supported by the total, far, intermediate, and near AUC values, which summarize optical performance across the full defocus range and facilitate comparison between different IOLs [14].

Contrast sensitivity, both under photopic and mesopic conditions, was well preserved. At 12 months, monocular and binocular contrast sensitivity values were within the range of age-matched normative data at all measured spatial frequencies, which suggests that the optical design of the Liberty IOL does not compromise contrast sensitivity, even in challenging lighting conditions. According to our results, Liberty IOL’s contrast sensitivity at high spatial frequencies turned out to be higher than a monofocal IOL from the same manufacturer [15].

Contrast sensitivity defocus curve showed the best performance at far distances, gradually decreasing towards intermediate, and achieving a secondary peak at near distances. A previous study also investigated the contrast sensitivity defocus curve of 677MY taken by the MLA application [16], but only the monocular performance. The significantly better contrast performance supported by CSV-1000 is suspected to be the result of differences in environmental lighting conditions, which are not reported in the studies.

Patient-reported outcomes indicate a favorable functional profile of the lens, characterized by low perceived difficulty in daily activities and a low incidence of visual disturbances. The high rates of spectacle independence across all distances, particularly for near and far vision, suggest that the optical design effectively meets patients’ visual demands in everyday life. Together, these findings reflect a high level of patient satisfaction and functional visual comfort. These current findings are consistent with previously published patient-reported outcomes for the Liberty trifocal IOL [17], supporting the reproducibility of its performance and its ability to deliver stable visual quality and patient satisfaction over time.

It is well established that PCO occurs more frequently after the implantation of hydrophilic IOLs than after hydrophobic designs [18–20]. In our study with the hydrophilic Liberty 677MTY, only 1.61% of the examined eyes (one case) required Nd: YAG capsulotomy—the standard treatment for clinically significant PCO—during the one-year follow-up period, accompanied by a PCO score of 3. Compared with literature data, the Liberty 677MTY has a markedly lower PCO-initiated Nd: YAG capsulotomy rate (1.61%) than the ~ 4% typically observed after hydrophilic IOL implantation [21]. This rate also falls within the lower range of hydrophobic lenses (1.73–4.5%) documented in large-scale clinical and registry cohorts [21–23]. This supports the hypothesis that when implanted in patients without elevated PCO risk, the hydrophilic Liberty lens demonstrates a safety profile comparable to that of hydrophobic IOLs.

This study benefits from a prospective design, standardized surgical technique, and a comprehensive evaluation of both objective and subjective outcomes. However, certain limitations should be acknowledged. The relatively small sample size prevents subgroup analyses, while the lack of a control group limits direct comparisons with other trifocal toric IOLs. Furthermore, the 12-month follow-up period, while sufficient for early and mid-term evaluation, does not capture late-onset complications. Finally, our study population also presented a lower mean age compared to the approximately 73-year average typically observed in patients undergoing IOL implantation [24, 25], which may have positively influenced the reported visual outcomes.

Overall, these findings contribute to the growing body of evidence supporting the use of trifocal toric IOLs in modern cataract surgery and underscore the value of monocular performance analysis in understanding lens-specific behavior. By highlighting both the visual performance and clinical reliability of the Liberty 677MTY, this study offers meaningful insights for surgeons seeking to optimize outcomes in patients with astigmatism and presbyopia.

## Conclusion

This prospective clinical investigation provides one of the first comprehensive evaluations of the Liberty 677MTY trifocal toric IOL, integrating real-life binocular visual experience with comprehensive monocular performance metrics in cataract patients with preoperative corneal astigmatism. The lens demonstrated excellent refractive predictability, robust rotational stability, and consistently strong visual acuity at all tested distances. Contrast sensitivity was well preserved under both photopic and mesopic conditions, exceeding age-matched normative values at higher spatial frequencies. Monocular defocus curve analysis confirmed the Liberty 677MTY’s classification as a Full Range of Field – Smooth (FRoF-Sm) IOL, supporting its ability to provide continuous functional vision across a wide range of distances. Patient-reported outcomes reflected high satisfaction levels, minimal visual disturbances, and strong spectacle independence for daily tasks. The safety profile was favorable, with a very low rate of posterior capsule opacification, indicating that the hydrophilic Liberty platform performs comparably to hydrophobic lenses when implanted in patients without elevated PCO risk.

By combining binocular patient experience with monocular optical characterization, this study offers a clearer understanding of the lens’s visual performance and clinical behavior. These results support the Liberty 677MTY as a reliable and effective option for visual rehabilitation in patients with both presbyopia and astigmatism.

## Data Availability

All the information and figures available for publication are contained in this manuscript. The raw data will not be disclosed. For more data/information related to the data, contact the corresponding author: Zoltán Zsolt Nagy, MD, PhD (nagyzzs100@gmail.com).

## Abbreviations

ACD: Anterior chamber depth
ANOVA: Analysis of variance
AUC: Area under the curve
AucF: Area under the curve – far
AucI: Area under the curve – intermediate
AucN: Area under the curve – near
AucT: Area under the curve - total
AXL: Axial length
CDVA: Corrected distance visual acuity
CSDC: Contrast sensitivity defocus curve
CYL: Cylindrical refraction
D: Diopter
DCIVA: Distance corrected intermediate visual acuity
DCNVA: Distance corrected near visual acuity
FRoF: Full range of field
FRoF-Sm: Full range of field - smooth
IOL: Intraocular lens
IOP: Intraocular pressure
logMAR: Logarithm of the minimum angle of resolution
MLA: Multifocal lens analyzer
OCT: Optical coherence tomography
OVD: Ophthalmic viscosurgical device
PCO: Posterior capsule opacification
RoF: Range of field
SD: Standard deviation
SE: Spherical equivalent refraction
SPH: Spherical refraction
UDVA: Uncorrected distance visual acuity
UIVA: Uncorrected intermediate visual acuity
UNVA: Uncorrected near visual acuity
VA: Visual acuity
VADC: Visual acuity defocus curve
